# Gastric Neuroendocrine Tumors and Pernicious Anemia: A Case Report and Literature Review

**DOI:** 10.7759/cureus.73553

**Published:** 2024-11-12

**Authors:** James J Rudolph, Obed Agyei, Talar Telvizian, Arezoo Ghaneie

**Affiliations:** 1 Internal Medicine, Pennsylvania Hospital, Philadelphia, USA; 2 Hematology and Medical Oncology, Lankenau Medical Center, Wynnewood, USA

**Keywords:** autoimmune atrophic gastritis, gnet, pernicous anemia, type 1 gastric neuroendocrine tumors, type 2 gastric neuroendocrine tumors, type 3 gastric neuroendocrine tumors

## Abstract

Gastrointestinal neuroendocrine tumors (GI-NETs) are rare neoplasms, with the gastric (stomach) subtype (G-NETs) representing a significant clinical focus. Type 1 G-NETs are particularly noteworthy due to their relationship with autoimmune atrophic gastritis (AAG) and pernicious anemia (PA), conditions that impact vitamin B12 absorption. This report presents the case of a patient with a type 1 G-NET identified at the initial diagnosis of PA, demonstrating the connection between these conditions. In the literature review, we discuss the general mechanisms underlying PA, including its etiology, pathogenesis, clinical presentations, and diagnostic approaches. Emphasis is placed on the importance of recognizing and diagnosing this condition early, given the treatable nature of the associated gastric neuroendocrine dysregulation. Additionally, the report examines the broad spectrum of G-NETs, with a special emphasis on the characteristics of type 1 tumors. By considering recent developments in the field, we provide an overview of the current understanding of G-NET epidemiology, classification, clinical features, diagnosis, and management strategies.

## Introduction

The journey toward understanding neuroendocrine tumors (NETs) commenced in 1868 with Rudolf Heidenhain's discovery of neuroendocrine cells, later noted by Nikolai Kulchitsky in 1897 [1]. However, it wasn't until 1907 that Siegfried Oberdorfer differentiated between carcinomas and less aggressive ileal lesions, termed "karzinoide" [2]. Despite initial skepticism, Oberdorfer's work gained acceptance, leading to further research. In 1914, Gosset and Masson identified carcinoids as endocrine-related tumors [3]. By 1929, Oberdorfer recognized their potential for metastasis [4]. Max Askanazy, a contemporary of Oberdorfer, discovered gastric involvement in carcinoid tumors [5]. In 1963, Williams and Sandler expanded the carcinoid definition and classified tumors by embryological origin (foregut, midgut, and hindgut) [6]. The World Health Organization (WHO) used "neuroendocrine tumor" to replace "carcinoid" in 2004 due to the latter's limitations. Today, we refer to these tumors as gastroenteropancreatic neuroendocrine tumors (GEP-NETs), highlighting their diverse cytokine and hormone secretion and the rarity of classic carcinoid symptoms. 

NETs are a heterogeneous category of tumors that can be benign or malignant. NETs are most commonly derived from neuroendocrine cells in the gastrointestinal and bronchopulmonary tracts; rarely, they occur in the breast, prostate, thymus, and skin [7]. “Neuroendocrine” describes widespread cells with both neurologic and endocrine features. The presence of dense core granules, which store monoamines similar to the serotonergic neurons of the CNS, depicts the “neuro” portion [8]. The production and release of those monoamines represent the “endocrine” portion. Roughly 40% of NETs are hormone-secreting. It is worth mentioning that neuroendocrine cells do not have axons or dendrites. 

The GI tract is the most common location for NETs (GI-NETs), accounting for approximately 50% of cases [9]. The literature describes GI-NETs as slow-growing and manifesting clinically with compressive mass effect, fibrosis, or symptoms from combinations of secreted components (histamine, serotonin, prostaglandins, etc.). When serotonin is the primary culprit, the latter is widely known as carcinoid syndrome. The classic presentation, co-occurring with hepatic metastasis, features dermal flushing, bronchospasm, gut-hypermobility, and hypotension. However, this is only seen in fewer than 10% of cases [10]. GI-NETs can develop in the small intestine (45%), rectum (20%), appendix (16%), colon (11%), and stomach (7%) [11]. While these data portray the stomach as one of the least common locations for GI-NETs, the incidence of these tumors is rapidly climbing. We will explore the epidemiology, histopathological grading, classification, clinical features, diagnosis, and management of gastric NETs (G-NETs), as well as the rare association with pernicious anemia through a case presentation.

## Case presentation

A 39-year-old male patient with no significant past medical history presented with ongoing generalized weakness, fatigue, and decreased appetite for two weeks, as well as an episode of non-bloody emesis just prior to arriving at the hospital. The patient's physical exam was unrevealing. Relevant laboratory tests and values are displayed in Table 1.

**Table 1:** Laboratory tests and values

Laboratory tests	Patient values	Reference range
Hemoglobin	6.2 g/dL	13.7 - 17.5 g/dL
Leukocyte count	4.84 × 10^9^/L	3.8 - 10.5 × 10^9^/L
Platelet count	82 × 10^9^/L	150 - 350 × 10^9^/L
Mean corpuscular volume	115 fL	83 - 98 fL
Total bilirubin	1.2 mg/dL	0.3 - 1.2 mg/dL
Bilirubin, direct	0.2 mg/dL	≤ 0.4 mg/dL
Ferritin	467 ng/mL	24 - 250 ng/mL
Iron saturation	49%	15 - 45%
Lactate dehydrogenase	4,191 IU/L	98 - 271 IU/L
Reticulocyte count	0.8 %	0.6 - 2.8 %
Haptoglobin	< 20 mg/dL	41 - 203 mg/dL
Folic acid	17.3 ng/mL	≥ 5.8 ng/mL
Vitamin B12	< 50 pg/mL	180 - 914 pg/mL
Methylmalonic acid	9,945 nmol/L	87 - 318 nmol/L
Intrinsic factor antibody	Positive	Negative
Parietal cell antibody	Positive	Negative
Parietal cell antibody titer	1:40 titer	< 1:20 titer
Gastrin	228 pg/mL	≤ 100 pg/mL
Chromogranin A	255 ng/mL	< 311 ng/mL

The patient was found to have macrocytic anemia, depicted by low hemoglobin and elevated mean corpuscular volume (MCV), as well as thrombocytopenia on complete blood count (CBC) testing. The patient was transfused two units of packed red blood cells and received 1000 mcg of intramuscular (IM) vitamin B12 before being admitted to the Internal Medicine service. Iron studies were assessed to rule out a potential deficiency contributing to the patient's anemia. Hemolysis labs were ordered to help determine the underlying cause of the patient's anemia, revealing a significantly elevated lactate dehydrogenase, low haptoglobin, and normal reticulocyte count. Folate and vitamin B12 levels were obtained in the setting of the patient's elevated MCV, showing low B12 and normal folate. Methylmalonic acid levels were then ordered, showing a profound elevation and prompting orders for intrinsic factor and parietal cell antibodies. After both autoantibodies returned positive, the patient’s clinical vignette indicated the diagnosis of pernicious anemia. Consultations were obtained with Gastroenterology and Hematology-Oncology for further workup and management. 

**Figure 1:**
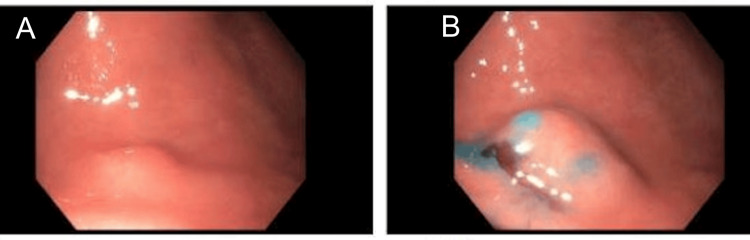
Upper endoscopic evaluation. Two views of the gastric antrum depict a single mucosal papule (nodule) (A, B). (B) Indigo carmine dye permits the lesion’s demarcations to become more discernible.

Per Hematology-Oncology, the patient was continued on IM vitamin B12 at 1000 mcg daily during the hospital course. An abdominal ultrasound was performed, demonstrating borderline splenomegaly. A blood smear showed marked macrocytic anemia with anisopoikliocytosis, nucleated red blood cells, and hypersegmented neutrophils. Gastroenterology services performed an endoscopic evaluation, which showed no evidence of mucosal bleeding and a single mucosal papule (nodule) in the gastric antrum as shown in Figure 1.

**Figure 2:**
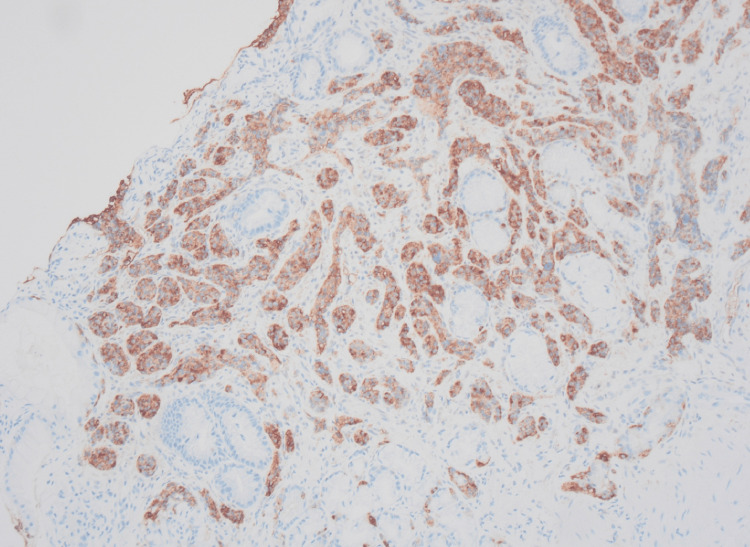
Sections of the antral nodule biopsy show infiltrating nests and cords of chromogranin-positive neuroendocrine cells; chromogranin immunohistochemical stain, 100x magnification

**Figure 3:**
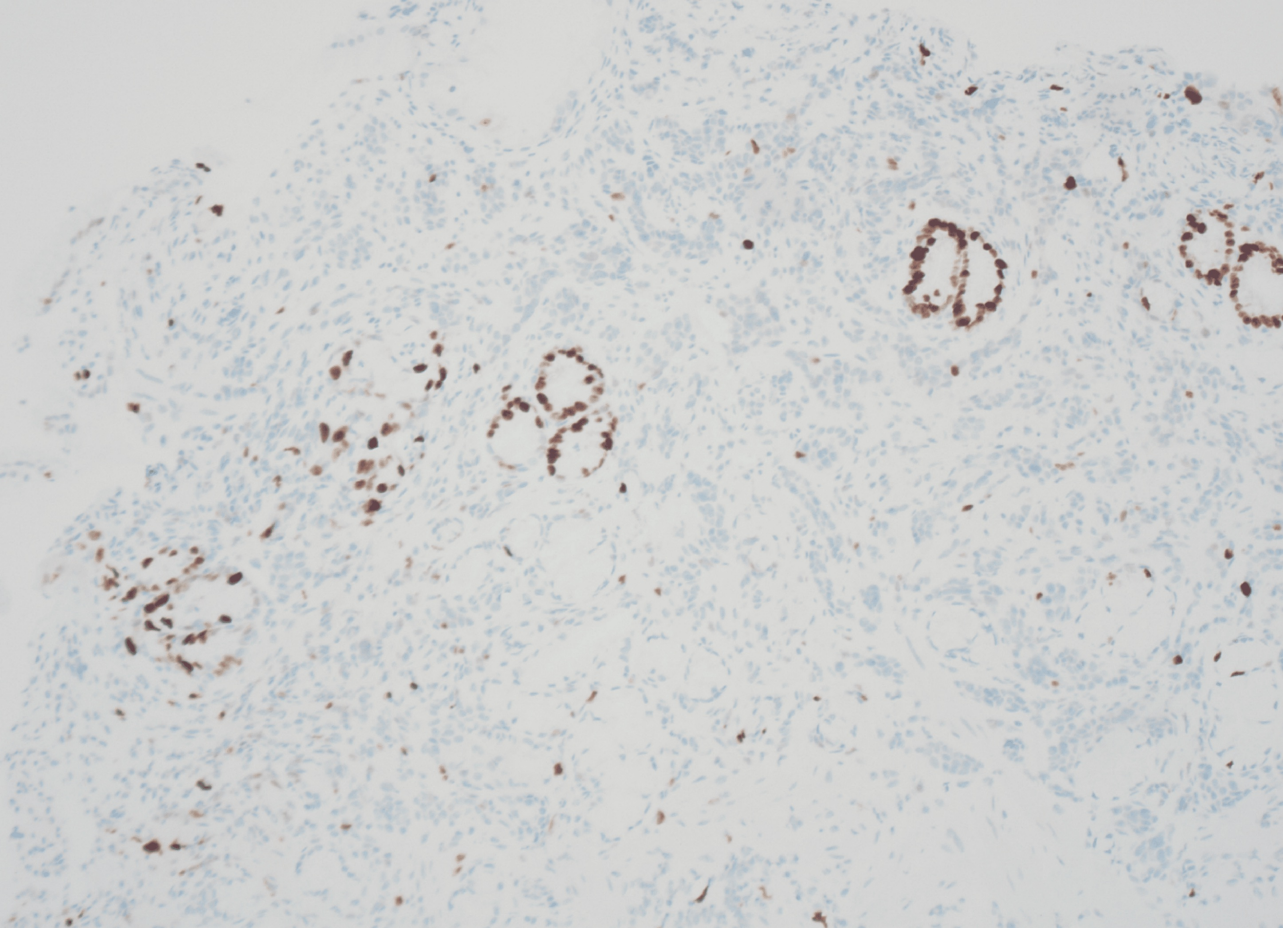
Sections of the antral nodule biopsy show a proliferation index of less than 3% and a mitotic count of less than 2/mm2; Ki-67 immunohistochemical stain, 100x magnification

Biopsies were obtained, and the samples were analyzed histologically. The biopsy from the gastric body showed changes of atrophic gastritis with loss of oxyntic cells as well as intestinal metaplasia with enterochromaffin-like cell hyperplasia demonstrated by chromogranin immunohistochemical stain. The biopsy from the gastric antrum nodule shows infiltrating nests and cords of chromogranin-positive neuroendocrine cells, displayed in Figure 2. Ki-67 immunohistochemical staining was performed with a proliferation rate below 3% and a mitotic count of less than 2/mm^2^, displayed in Figure 3. With these findings, the diagnosis of a well-differentiated NET (Grade 1) was made.

Serum gastrin and chromogranin levels were subsequently obtained, showing an elevation in gastrin and normal chromogranin levels. The patient underwent a CT chest, abdomen, and pelvis with IV contrast to evaluate for thymoma and further staging/metastatic workup. The CT scan showed no evidence of metastatic disease. Upon continued improvement in the patient's symptoms and lab values, the patient was discharged with instructions to continue 1000 mcg of IM vitamin B12 daily for a total of seven days, then once weekly for four weeks, then once monthly for life. The patient was scheduled with outpatient Hematology-Oncology for routine CBC with differential and vitamin B12 levels during treatment. The patient's laboratory values, including hemoglobin, normalized during follow-up visits at three and six months post hospitalization.

## Discussion

Epidemiology

NETs constitute only 0.5% of all malignant conditions and 2% of all malignant tumors of the GI [12]. Utilizing the National Cancer Institute (NCI)’s Surveillance, Epidemiology, and End Results (SEER) database, the most extensive retrospective population-based analysis of NETs identified 64,971 cases [12]. This 2017 study found a 6.4-fold increase in annual age-adjusted incidence of NETs in all organs from 1973 (1.09-100,000 persons) to 2012 (6.98 per 100,000 persons). GEP-NETs exhibited the highest increase in incidence among primary tumor sites, rising from one case per 100,000 to 3.56 cases per 100,000. The highest proportional change occurred in rectal NETs and G-NETs [13].

The role of G-NETs in this overall increase in GEP-NETs is better understood by a 2020 retrospective study on patients in the SEER database diagnosed with G-NETs from 1975 to 2016 [14]. The age-adjusted incidence of G-NETs increased from 0.309 to 6.149 per 1,000,000 persons, nearly a 20-fold increase in the past four decades. This increase in incidence is partially due to advanced histological ability and more frequent use of diagnostic tools such as endoscopy.

Most commonly, G-NETs arise from the enterochromaffin-like (ECL) cells found in the gastric mucosa. Specifically, ECL cells are located in the oxyntic mucosa and mobilize histamine to the parietal cells, regulating hydrogen chloride (HCl) secretion [15].

Histopathological grading 

In 2019, the WHO introduced a new classification of GI-NETs, dividing gastrointestinal neuroendocrine neoplasms (NENs) into NETs and neuroendocrine carcinomas (NECs) based on molecular differences. Mutations in *MEN1*, *DAXX*, and *ATRX* are defining features for well-differentiated NETs, while NECs commonly have TP53 or RB1 mutations [16,17]. NENs are broadly divided into two categories: well and poorly differentiated. Well-differentiated NENs, or NETs, are further divided into histological grades one to three using the mitotic and Ki-67 indices. Poorly differentiated NENs are categorized as NECs, which are further divided into small and large cell types [16,17]. Finally, there is a third category with variable differentiation, grade, as well as mitotic and Ki-67 indices. These are referred to as mixed neuroendocrine-non-neuroendocrine neoplasm (MiNEN) (previously mixed adenoneuroendocrine carcinoma). The 2019 WHO classification of GI-NETs is outlined in Table 2.

**Table 2:** WHO 2019 classification of GI-NETs NET: neuroendocrine tumor; SCNEC: small cell type neuroendocrine carcinoma; LCNEC: large cell type neuroendocrine carcinoma; MiNEN: mixed neuroendocrine-non-neuroendocrine neoplasm; GI-NET: gastrointestinal neuroendocrine tumors Reference: [16,17]

Terminology	Differentiation	Grade	Mitotic rate (mitoses/mm^2^)	Ki-67 index (%)
Grade one NET	Well-differentiated	Low	<2	<3
Grade two NET	Well-differentiated	Intermediate	2-20	3-20
Grade three NET	Well-differentiated	High	>20	>20
SCNEC	Poorly differentiated	High	>20	>20
NEC, large cell type (LCNEC)	Poorly differentiated	High	>20	>20
MiNEN	Well or poorly differentiated	Variable	Variable	Variable

Classification and clinical features

G-NETs can be subdivided into three types, each with specific clinical features, as organized in Table 3. 

**Table 3:** Three types of G-NETs with respective characteristics MEN1: multiple endocrine neoplasia type 1; ZES: Zollinger-Ellison syndrome; G-NET: gastrointestinal neuroendocrine tumors Reference: [9,18]

Characteristic	Type-1 G-NETs	Type-2 G-NETs	Type-3 G-NETs
Prevalence	70-80%	5-10%	15-20%
Etiology	Chronic atrophic gastritis	MEN1/ZES	Sporadic
Gender preference	women > men	men = women	men > women
Locational preference	Fundus/body	Fundus/body	Variable
Quantity of lesions	multiple	multiple	solitary
Tumor size (avg.)	10-20 mm	~10 mm	>20 mm
Serum gastrin	High	High	Normal
Gastric pH	High	Low	Normal
Metastatic potential	Very low	Low-moderate	High
Prognosis	Excellent	Good	Poor

Type-1 G-NETs

Type-1 G-NETs are the most common presenting type (70-80%) and have a strong female preponderance [19-21]. The favored hypothesis entails ECL cell development into carcinoids due to chronic stimulation by high gastrin levels. This is often seen in patients with achlorhydria associated with AAG secondary to pernicious anemia or chronic *Helicobacter pylori* infections [22]. When gastric acid secretion is impeded, hyperplasia of the G-cells occurs, and gastrin hypersecretion results. Gastrin then acts on cholecystokinin-2 receptors, which are densely supplied within ECL cells, facilitating the proliferation and development of type-1 G-NETs [16,23].

Type-1 G-NETs are often diagnosed incidentally during the endoscopic evaluation of patients with dyspepsia or anemia. Grossly, they appear as multiple sub-centimeter polypoid lesions, with or without central ulcerations, in the gastric body and fundus [9,24]. Gastric pH is elevated due to a loss of HCl-secreting parietal cells and can be useful in cases where gastric atrophy is not obvious [25]. Elevations in serum chromogranin A (CgA) support the diagnosis over elevated serum gastrin, as AAG commonly occurs without the presence of type-1 G-NETs. Histologically, G-NETs are often positive for CgA, Neuron-specific enolase, and vesicular monoamine transporter two [9].

Endoscopic ultrasound (EUS) has proven to be a useful tool in assessing and treating G-NETs. EUS can effectively stage localized G-NETs, often identifying patients who can safely undergo endoscopic mucosal resection (EMR) [26]. Computerized tomography (CT) with contrast or magnetic resonance imaging (MRI) is required to rule out metastatic disease, although this is rare with type-1 G-NETs (2-5% of cases). 

Endoscopic evaluation and resection are the mainstay of treatment for type 1 G-NETs, largely dependent on the lesions' size, depth, and quantity. Lesions <10 mm can be monitored annually or endoscopically removed. Lesions that are not extensive and >10 mm can be resected via polypectomy, EMR, or endoscopic submucosal dissection (ESD) for larger lesions resistant to EMR [25,27]. Patients with less than or equal to six lesions >20 mm in size should be individualized and can undergo endoscopic resection or be considered for surgical resection [27,28]. Surgical resection is recommended for advanced lesions >10 mm, with involvement of the muscularis propria and/or local lymph nodes as assessed by EUS. Gastric antrectomy is an option for patients with multifocal disease (>six lesions, three to four lesions >10 mm, or one lesion >20 mm), invasive disease, or recurrent disease [9,19,25,27].

Antrectomy effectively resolves hypergastrinemia mediated by G cells, leading to regression of type-1 G-NET lesions in over 90% of cases [25]. Patients should undergo surveillance endoscopy at six-month intervals after endoscopic resection or surgery. Although type-1 G-NETs are recurring tumors (median time 24 months), the prognosis remains excellent, with a five-year survival of 90-95% [29]. Pharmacotherapy options are limited; however, somatostatin analogs (i.e., octreotide) can be used in the setting of recurrence following resection or multiple lesions not amenable to resection by reducing serum gastrin [30]. Additionally, netazepide, a receptor antagonist for gastrin/cholecystokinin-2, has demonstrated efficacy in diminishing the size and quantity of type-1 G-NETs, alongside lowering plasma chromogranin A levels [17,30]. Further large-scale and randomized studies are needed to determine netazepide efficacy.

Type-2 G-NETs

Type-2 G-NETs are the least common subtype (5-10%) and usually occur in response to gastrinomas (gastrin-producing tumors), also termed Zollinger Ellison Syndrome (ZES), associated with MEN1. These occur equally in male and female patients [19]. Hypergastrinemia causes hypertrophy of the gastric mucosa, ECL hyperplasia, and subsequent ECL dysplasia. Interestingly, sporadic ZES is far more common (70-80%) than ZES associated with MEN1 (20-30%) [18,31]. However, type-2 G-NETs occur in <1% of sporadic ZES and 13-43% of MEN1-ZES [9]. This is thought to be due to the mutation of the *MEN1* gene, located on chromosome 11q13, which results in a defect in the tumor suppressor protein menin. 

As with all G-NETs, diagnostic confirmation is achieved via endoscopy, demonstrating multiple, small, polypoid lesions in the stomach. Like type-1 G-NETs, serum gastrin levels will be elevated; however, gastric pH is low (<two) secondary to hyperchlorhydria [18]. Early diagnosis is a prognostic factor in MEN1 patients and biochemical identification of hypergastrinemia has been shown to recognize the presence of gastrinomas before they are seen on endoscopy [32]. This emphasizes the utility of the secretin stimulation test, in which serial elevations in gastrin after secretin administration constitute a positive test. Identifying the other components of the MEN1 syndrome can also aid in diagnosis, including a workup of pituitary and parathyroid hormones as well as serum calcium levels. 

Metastasis occurs in 10-30% of patients at presentation and the prognosis is good, with a five-year survival rate of 70-90% [9,19,33]. Treatment is similar to that of type-1 G-NETs, with the addition of gastrinoma resection or, in extensive cases, gastrectomy [30].

Type-3 G-NETs

Type-3 G-NETs encompass 15-20% of all G-NETs and lack associated conditions. These tumors are heterogeneous in nature. They demonstrate the highest risk for metastasis (>50%), often at initial diagnosis, favoring the liver and regional lymph nodes [9]. There is a strong male predominance (>50 years of age) with a 2.8/1 male-to-female ratio [30]. The updated WHO 2019 pathological classification is used to gauge biological behavior and prognosis [34]. They are most commonly derived from ECL cells; however, hyperplasia is absent, and they lack gastrin dependence. 

Endoscopy typically reveals a solitary, polypoid, often large lesion (>2cm) favoring the gastric antrum [17,19,35]. Histologically, these lesions are classified as G3-NECs. Common presenting symptoms include those of gastric cancer, anemia, decreased appetite, dyspepsia, and weight loss. The classic presentation of carcinoid syndrome is rare in patients with G-NETs (<1%) and is exclusive to type-3 G-NETs that have metastasized to the liver [18,19].

The cornerstone of treatment for non-metastatic type-3 G-NETs has been gastrectomy with regional lymphadenectomy on the basis of lymph node metastasis (LNM). However, small retrospective studies have recently shown that endoscopic resection of type-3 G-NETs ≤10 mm, limited to the mucosa or submucosa, and with a grade of G1 (Ki-67 <3% and mitotic rate <2 mitoses/mm^2^) have demonstrated exceptional survival outcomes despite the risk of LNM [36-38]. For metastatic disease, treatments include octreotide (for carcinoid syndrome) as well as systemic chemotherapy, molecular targeted agents, targeted radionucleotide therapies, transarterial chemoembolization (TACE), and radiofrequency ablation (for hepatic metastasis) [9]. Despite aggressive intervention, type-3 G-NETs carry the worst prognosis, with a five-year survival rate of less than 35% [34,35]. Fluorodeoxyglucose-positron emission tomography (FGD-PET) scans are indicated for the diagnosis and surveillance of metastasis in type-3 G-NETs [39]. However, the utility of FGD-PET scans for low to intermediate-grade G-NETs (types 1 and 2) is still under debate [40].

For completeness, it is necessary to highlight that there has been a selection of studies addressing the possibility of a fourth type of G-NET. In current literature, type-4 G-NETs are described as being the most rare subtype, carrying the worst prognosis, and occurring in males > 60 years of age. They are highly aggressive, often classified as G3-NECs, and closely resemble gastric adenocarcinomas [17]. Further investigation is needed to assess these lesions as they are not included in the most recent WHO classifications. 

Pernicious anemia, autoimmune gastritis, and type-1 G-NETs

Pernicious anemia is autoimmune gastritis caused by antibodies directed at parietal cells, intrinsic factors, or both, causing a malabsorptive deficiency of vitamin B12 (cobalamin). The prevalence in the general population is 0.1% and 1.9% in those over 60 years of age [41]. It is worth mentioning that *H. pylori* infection and its relationship to AAG is still under investigation, as *H. pylori*-positive patients have shown to have higher titers of anti-parietal cell antibodies, which decrease after *H. pylori* eradication [42]. Patients with AAG are often asymptomatic but can present with dyspepsia, fatigue, weakness, and neurological disturbances. Megaloblastic anemia is characteristic and iron deficiency is not uncommon [43].

The diagnosis of AAG is based on serology profile and histology of gastric biopsies. The sensitivity and specificity of anti-parietal cell antibodies for the diagnosis of AAG are 81% and 90%, respectively, while anti-intrinsic factor antibody carries a low sensitivity (27%) and high specificity (100%) [42]. Serum gastrin and chromogranin levels are typically elevated in these patients. However, the latter can be observed in several other conditions and is not used to confirm diagnosis. Suspicion of AAG mandates endoscopic evaluation, which demonstrates mucosal erythema and nodularity. Histologic evaluation of gastric biopsies is the gold standard for the diagnosis of AAG [44]. Histologic features include destruction of oxyntic mucosa, hyperplasia of gastrin G cells, and hyperplasia of ECL cells. 

In 2019, a pivotal study by Lenti et al. investigated diagnostic delay in AAG [45]. In a 10-year period, 291 patients with AAG (mean age at diagnosis 61±15 years; female-to-male ratio 2.3/1) were found to have a median overall diagnostic delay of 14 months. The overall delay in 54.3% of patients was ≥12 months, for 37.5%, ≥24 months, and in 28.3%, ≥36 months [46]. Half of these patients had gastrointestinal symptoms (reflux, dyspepsia, abdominal pain, diarrhea, and weight loss), one-third had hematological abnormalities (anisocytosis and macrocytic anemia), and some had symptoms associated with autoimmune disorders. This study underscores the need for proactive diagnostic measures with serology, MCV, hemoglobin, and gastrin levels. 

The incidence and prevalence of type-1 G-NETs in patients with AAG have steadily increased, estimated at 2.8-7% and 1-12.5%, respectively [42,47-49]. Despite variation in selection criteria and scarcity of observational studies assessing type-1 GNETs, current data suggests the benefit of routine endoscopic screening at the initial time of PA/AAG diagnosis. This is highlighted by the American Gastroenterology Association’s (AGA) Clinical Practice Update Expert Review, which includes the recommendation for providers to recognize PA as a late-stage manifestation of AAG and to employ endoscopy with topographical biopsies at the initial diagnosis for risk stratification in addition to ruling out gastric neoplasias (including G-NETs) [50].

## Conclusions

G-NETs encompass a diverse range of tumors, with type-1 G-NETs being the most common. These tumors are often associated with conditions like autoimmune gastritis and pernicious anemia and are typically detected during routine endoscopic evaluations. The prognosis for type-1 G-NETs is generally favorable, with management focused on removal and regular monitoring. Other subtypes, such as type-2 and type-3 G-NETs, present more complex challenges due to their potential for metastasis and varying treatment requirements.

Our case highlights the importance of early detection and vigilant screening in managing G-NETs, particularly in patients with related conditions. Ensuring that patients receive appropriate screening can lead to timely diagnoses and better outcomes. As understanding of G-NETs evolves, continued research and improvements in clinical practices will be key to optimizing patient care and outcomes.

## References

[1] Drozdov I, Modlin IM, Kidd M, Goloubinov VV (2009). From Leningrad to London: the saga of Kulchitsky and the legacy of the enterochromaffin cell. Neuroendocrinology.

[2] Modlin IM, Shapiro MD, Kidd M (2004). Siegfried Oberndorfer: origins and perspectives of carcinoid tumors. Hum Pathol.

[3] Tsoucalas G, Karamanou M, Androutsos G (2011). The eminent German pathologist Siegfried Oberndorfer (1876-1944) and his landmark work on carcinoid tumors. Ann Gastroenterol.

[4] Gosset A, Masson P (1914). Endocrine tumors of the appendix [Article in French]. Presse Med.

[5] Klöppel G, Dege K, Remmele W, Kapran Y, Tuzlali S, Modlin IM (2007). Siegfried Oberndorfer: a tribute to his work and life between Munich, Kiel, Geneva, and Istanbul. Virchows Arch.

[6] Hallet J, Law CH, Cukier M, Saskin R, Liu N, Singh S (2015). Exploring the rising incidence of neuroendocrine tumors: a population-based analysis of epidemiology, metastatic presentation, and outcomes. Cancer.

[7] Kwon DH, Nakakura EK, Bergsland EK, Dai SC (2017). Gastric neuroendocrine tumors: management and challenges. Gastrointestinal Cancer: Targets and Therapy.

[8] Oronsky B, Ma PC, Morgensztern D, Carter CA (2017). Nothing but NET: a review of neuroendocrine tumors and carcinomas. Neoplasia.

[9] Ahmed M (2020). Gastrointestinal neuroendocrine tumors in 2020. World J Gastrointest Oncol.

[10] Modlin IM, Kidd M, Latich I, Zikusoka MN, Shapiro MD (2005). Current status of gastrointestinal carcinoids. Gastroenterology.

[11] Maggard MA, O'Connell JB, Ko CY (2004). Updated population-based review of carcinoid tumors. Ann Surg.

[12] Dasari A, Shen C, Halperin D (2017). Trends in the incidence, prevalence, and survival outcomes in patients with neuroendocrine tumors in the United States. JAMA Oncol.

[13] Lawrence B, Gustafsson BI, Chan A, Svejda B, Kidd M, Modlin IM (2011). The epidemiology of gastroenteropancreatic neuroendocrine tumors. Endocrinol Metab Clin North Am.

[14] Hu P, Bai J, Liu M (2020). Trends of incidence and prognosis of gastric neuroendocrine neoplasms: a study based on SEER and our multicenter research. Gastric Cancer.

[15] Lindström E, Chen D, Norlén P, Andersson K, Håkanson R (2001). Control of gastric acid secretion: the gastrin-ECL cell-parietal cell axis. Comp Biochem Physiol A Mol Integr Physiol.

[16] Nagtegaal ID, Odze RD, Klimstra D (2020). The 2019 WHO classification of tumours of the digestive system. Histopathology.

[17] Matsui K, Jin XM, Kitagawa M, Miwa A (1998). Clinicopathologic features of neuroendocrine carcinomas of the stomach: appraisal of small cell and large cell variants. Arch Pathol Lab Med.

[18] Roberto GA, Rodrigues CM, Peixoto RD, Younes RN (2020). Gastric neuroendocrine tumor: A practical literature review. World J Gastrointest Oncol.

[19] Sato Y, Hashimoto S, Mizuno K, Takeuchi M, Terai S (2016). Management of gastric and duodenal neuroendocrine tumors. World J Gastroenterol.

[20] Kargwal N, Panda V, Jha A, Singh CB (2021). Gastric neuroendocrine tumor. Surg J (N Y).

[21] Wardlaw R, Smith JW (2008). Gastric carcinoid tumors. Ochsner J.

[22] Sato Y, Iwafuchi M, Ueki J (2002). Gastric carcinoid tumors without autoimmune gastritis in Japan: a relationship with Helicobacter pylori infection. Dig Dis Sci.

[23] Avila J, Reyes I, Villacrés L (2020). Pernicious anemia and gastric carcinoid tumor: a case report and literature review. Am J Med Case Rep.

[24] Sato Y (2015). Endoscopic diagnosis and management of type I neuroendocrine tumors. World J Gastrointest Endosc.

[25] Henderson-Jackson E, Sheikh U, Muhammad J, Coppola D, Nasir A (2016). Neuroendocrine neoplasms of the stomach. Neuroendocrine Tumors: Review of Pathology, Molecular and Therapeutic Advances.

[26] Kim MK (2012). Endoscopic ultrasound in gastroenteropancreatic neuroendocrine tumors. Gut Liver.

[27] Gluckman CR, Metz DC (2019). Gastric neuroendocrine tumors (carcinoids). Curr Gastroenterol Rep.

[28] Kunz PL, Reidy-Lagunes D, Anthony LB (2013). Consensus guidelines for the management and treatment of neuroendocrine tumors. Pancreas.

[29] Wang M, Cheng S, Zhu L (2022). Metastasis prevalence and survival of patients with T1-2 gastric neuroendocrine tumor treated with endoscopic therapy and surgery. Dig Dis Sci.

[30] Sok C, Ajay PS, Tsagkalidis V, Kooby DA, Shah MM (2024). Management of gastric neuroendocrine tumors: a review. Ann Surg Oncol.

[31] Massironi S, Rossi RE, Laffusa A (2023). Sporadic and MEN1-related gastrinoma and Zollinger-Ellison syndrome: differences in clinical characteristics and survival outcomes. J Endocrinol Invest.

[32] Giusti F, Cioppi F, Fossi C, Marini F, Masi L, Tonelli F, Brandi ML (2022). Secretin stimulation test and early diagnosis of gastrinoma in MEN1 syndrome: survey on the MEN1 florentine database. J Clin Endocrinol Metab.

[33] Laffi A, Lania AG, Ragni A (2023). Gastric neuroendocrine tumors (g-NETs): a systematic review of the management and outcomes of type 3 g-NETs. Cancers (Basel).

[34] Li YL, Qiu XD, Chen J (2020). Clinicopathological characteristics and prognosis of 77 cases with type 3 gastric neuroendocrine tumours. World J Gastrointest Oncol.

[35] Chung CS, Tsai CL, Chu YY (2018). Clinical features and outcomes of gastric neuroendocrine tumors after endoscopic diagnosis and treatment: a Digestive Endoscopy Society of Tawian (DEST). Medicine (Baltimore).

[36] Hirasawa T, Yamamoto N, Sano T (2021). Is endoscopic resection appropriate for type 3 gastric neuroendocrine tumors? Retrospective multicenter study. Dig Endosc.

[37] Panzuto F, Ramage J, Pritchard DM (2023). European Neuroendocrine Tumor Society (ENETS) 2023 guidance paper for gastroduodenal neuroendocrine tumours (NETs) G1-G3. J Neuroendocrinol.

[38] Min BH, Hong M, Lee JH (2018). Clinicopathological features and outcome of type 3 gastric neuroendocrine tumours. Br J Surg.

[39] Deroose CM, Hindié E, Kebebew E, Goichot B, Pacak K, Taïeb D, Imperiale A (2016). Molecular imaging of gastroenteropancreatic neuroendocrine tumors: current status and future directions. J Nucl Med.

[40] Rufini V, Calcagni ML, Baum RP (2006). Imaging of neuroendocrine tumors. Semin Nucl Med.

[41] Andres E, Serraj K (2012). Optimal management of pernicious anemia. J Blood Med.

[42] Castellana C, Eusebi LH, Dajti E (2024). Autoimmune atrophic gastritis: A clinical review. Cancers (Basel).

[43] Carmel R, Weiner JM, Johnson CS (1987). Iron deficiency occurs frequently in patients with pernicious anemia. JAMA.

[44] Rodriguez-Castro KI, Franceschi M, Miraglia C (2018). Autoimmune diseases in autoimmune atrophic gastritis. Acta Biomed.

[45] Lenti MV, Miceli E, Cococcia S (2019). Determinants of diagnostic delay in autoimmune atrophic gastritis. Aliment Pharmacol Ther.

[46] Walker MM (2019). Editorial: determinants of diagnostic delay in autoimmune atrophic gastritis-a salutary lesson. Aliment Pharmacol Ther.

[47] Nehme F, Rowe K, Palko W, Tofteland N, Salyers W (2020). Autoimmune metaplastic atrophic gastritis and association with neuroendocrine tumors of the stomach. Clin J Gastroenterol.

[48] Modlin IM, Lye KD, Kidd M (2003). Carcinoid tumors of the stomach. Surg Oncol.

[49] Massironi S, Gallo C, Elvevi A, Stegagnini M, Coltro LA, Invernizzi P (2023). Incidence and prevalence of gastric neuroendocrine tumors in patients with chronic atrophic autoimmune gastritis. World J Gastrointest Oncol.

[50] Shah SC, Piazuelo MB, Kuipers EJ, Li D (2021). AGA clinical practice update on the diagnosis and management of atrophic gastritis: expert review. Gastroenterology.

